# MEF2 plays a significant role in the tumor inhibitory mechanism of encapsulated RENCA cells via EGF receptor signaling in target tumor cells

**DOI:** 10.1186/s12885-018-5128-5

**Published:** 2018-12-04

**Authors:** Prithy C. Martis, Atira T. Dudley, Melissa A. Bemrose, Hunter L. Gazda, Barry H. Smith, Lawrence S. Gazda

**Affiliations:** 1 The Rogosin Institute-Xenia Division, 740 Birch Road, Xenia, OH 45385 USA; 2 The Rogosin Institute, New York, NY 10021 USA; 3 NewYork-Presbyterian Hospital and Weill Medical College of Cornell University, New York, NY 10021 USA

**Keywords:** Tumor inhibition, Cell therapy, MEF2, EGFR

## Abstract

**Background:**

Agarose encapsulated murine renal adenocarcinoma cells (RENCA macrobeads) are currently being investigated in clinical trials as a treatment for therapy-resistant metastatic colorectal cancer. We have previously demonstrated the capacity of RENCA macrobeads to produce diffusible substances that markedly inhibit the proliferation of epithelial-derived tumor cells outside the macrobead environment. This study examined the molecular mechanisms underlying the observed inhibition in targeted tumor cells exposed to RENCA macrobeads.

**Methods:**

We evaluated changes in transcription factor responses, participating intracellular signaling pathways and the involvement of specific cellular receptors in targeted tumor cells exposed to RENCA macrobeads.

**Results:**

Factors secreted by RENCA macrobeads significantly up-regulated the activity of the MEF2 transcription factor as well as altered the transcription of *MEF2b* and *MEF2d* isoforms in targeted tumor cells. Suppression of individual or multiple MEF2 isoforms in target tumor cells markedly reduced the growth inhibitory effects of RENCA macrobeads. Furthermore, these effects were linked to the activation of the EGF receptor as attenuation of EGFR resulted in a substantial reduction of the cancer cell growth-inhibitory effect.

**Conclusions:**

Since interruption of the EGFR signaling cascade did not eliminate RENCA macrobead-induced growth control, our data suggests that RENCA macrobeads exert their full growth inhibitory effects through the simultaneous activation of multiple signaling pathways. In contrast to a precision medicine approach targeting single molecular abnormalities, the RENCA macrobead functions as a biological-systems therapy to re-establish regulation in a highly dysfunctional and dysregulated cancer system.

**Electronic supplementary material:**

The online version of this article (10.1186/s12885-018-5128-5) contains supplementary material, which is available to authorized users.

## Background

Cancer caused by genetic mutations and epigenetic changes has been thought to arise as clonal growth from a single founder cell [1]. However, it is becoming increasingly apparent that cancer is a disease of significant genetic diversity, both between patients and among cancer cells within a single patient’s tumor [2]. Conventional approaches to eliminate cancer cells (e.g. surgical resection, radiation and chemotherapy) and more recently, immunotherapy, have reported some success [3–6], but these benefits have only been realized in a minority of cancers. More concerning is the fact that greater than 90% of deaths from cancer result from the metastatic spread of the original tumor to a vital organ or system [7]. Our attempts to eliminate all unregulated tumor cells are failing for the majority of cancers and thus some consideration into alternatives to the challenges of complete tumor cell eradication is warranted.

To this end, we have previously demonstrated a remarkable tumor growth regulatory network that exists between different cancers, and even between species [8]. We have reported on the ability of agarose encapsulated murine renal adenocarcinoma cells (RENCA macrobeads) to form tumor colonies that survive indefinitely in vitro while maintaining a given colony size. Co-culture of the RENCA macrobeads (or conditioned media from the macrobeads) with freely growing cancer cells results in growth inhibition of the non-encapsulated cancer cells. The macrobeads secrete numerous proteins that diffuse out of the agarose. Many of these proteins have known tumor inhibitory capabilities. Importantly, the encapsulated RENCA cells demonstrate growth inhibition of not only murine cancer cells but also human cancer cell lines. Because many of the identified secreted proteins maintain significant species sequence conservation, we have postulated that these proteins can interact with target tumor cells from other species and produce growth inhibition [8]; although the molecular mechanism(s) of this inhibition is not known.

Given the vast number of macrobead-secreted proteins and therefore the difficulty of identifying the role of individual proteins in the observed tumor inhibition, the response of the target tumor cells was examined in the studies reported herein. Because transcription factors such as p53 are perhaps the most deregulated control mechanisms in all of cancer [9, 10], we first focused on changes in transcription factor responses of tumor cells exposed to RENCA macrobeads. We identified a significant up-regulation in the activity of the transcription factor myocyte-enhancer factor 2 (MEF2) and subsequently worked upstream to identify participating intracellular signaling pathways, and finally the role of specific cellular receptors. In this paper, we report on one intracellular signaling pathway, acting through the epidermal growth factor receptor (EGFR) and the transcription factor MEF2 that is at least partially responsible for the observed growth inhibition induced by exposure to RENCA macrobeads.

## Methods

### Cell lines and culture media

The RENCA tumor cell line used for these experiments is a renal cortical adenocarcinoma that arose spontaneously in Balb/c mice, originally obtained from the National Cancer Institute (Bethesda, MD) and now available from ATCC (American Type Culture Collection, Manassas, VA, CRL-2947) as previously described [8]. The cell line was authenticated based on morphology, isoenzymology and/or Cytochrome C subunit I (COI) PCR assays. RENCA cells were maintained in vitro (5% CO_2_ + air at 37 °C) in tissue culture flasks (Greiner Bio-One, Monroe, NC) containing RPMI 1640 (Life Technologies, Carlsbad, CA) with 10% newborn calf serum (NCS; Life Technologies). DU145 (human prostate carcinoma), originally obtained from ATCC (HTB-81) was cultured in RPMI 1640 supplemented with 10% fetal bovine serum (FBS; Life Technologies). DU145 cells were authenticated based on viability, recovery, growth, morphology and isoenzymology by the supplier. Cell passages were limited to no more than 20 from a frozen stock of these cells unless otherwise indicated. Routine testing for *Mycoplasma* contamination has been consistently negative (Bionique Testing Laboratories, Saranac Lake, NY). RENCA macrobeads were prepared as previously described [8, 11]. Briefly, 1.5 × 10^5^ RENCA cells were mixed with 100 μL of 0.8% agarose (HSB-LV; Lonza Copenhagen ApS, Vallensbak Strand) in MEM and expelled into mineral oil to form the core of the macrobead. Following washing with RPMI 1640, the core was rolled in approximately 1 mL of 4.5% agarose to apply an outer coat. RENCA macrobeads were cultured in 90-mm Petri dishes (Nunc, Rochester, NY) at 10 macrobeads per 40 mL of RPMI 1640 supplemented with 10% NCS for use with RENCA cells or 10% FBS for assays using DU145 cells. Conditioned media was collected after 5 days of culture with RENCA macrobeads. Medium was refreshed weekly. RENCA macrobeads used in experiments were greater than 18 weeks of age unless otherwise specified.

### Cignal reporter assay

For the 45-pathway Cignal reporter assay (SABiosciences, Frederick, MD) and the Cignal MEF2 reporter assay (SABiosciences), 10,000 RENCA cells were reverse transfected with pathway-focused transcription factor-responsive luciferase reporters or control constructs using Lipofectamine 2000 or 3000 (Life Technologies). Transiently transfected RENCA cells were exposed to naïve or 5-day conditioned media from RENCA macrobeads for 24 h. Regulation of each reporter was measured using the dual-luciferase reporter assay (Promega, Madison, WI) on a Synergy 2 microplate reader (Bio-Tek, Winooski, VT). Luminescence values for the experimental reporter signal (firefly luciferase, FL) and the internal control signal (Renilla luciferase, RL) were expressed as ratios (FL/RL) to correct for variations in transfection efficiency and cell number. Fold change in relative luciferase units (RLUs) was calculated based on normalized luciferase activity of the conditioned media response relative to the naïve media response. Each experiment was performed in triplicate at minimum.

### RNA isolation and gene expression measurement by qRT-PCR

Total RNA was isolated from RENCA, DU145, and DU145/GR cells cultured in naïve media or together with RENCA macrobeads as previously described [12]. Briefly, RNA was extracted using a RNeasy mini kit followed by genomic DNA elimination with RNase-Free DNase (Qiagen, Valencia, CA) according to manufacturer’s recommendations. RNA concentration and quality was determined using the Agilent 2100 RNA Bioanalyzer with the Agilent 6000 Nano Kit (Agilent Technologies, Santa Clara, CA). To confirm RNA quality, electropherograms were evaluated where purified RNA had a RNA Integrity Number (RIN) between 9.2 and 10. For quantitative real-time PCR (qRT-PCR), RNA (500 ng) was reverse transcribed using the RT^2^ First Strand Kit (Qiagen). Synthesized cDNA (20 ng) was combined with 2X TaqMan® Gene Expression Master Mix, 250 nM 6- FAM™ dye labeled TaqMan® MGB probe, and 900 nM each of forward and reverse unlabeled primers for *MEF2A*, *MEF2B*, *MEF2C*, *MEF2D*, and the housekeeping genes, *GAPDH* and *TBP* (IDT, Coralville, IA). The primer and probe sequences used in this study are included in Tables 1 and 2 for samples of mouse and human origin respectively. Each reaction was initially incubated at 50 °C for 2 min and 95 °C for 10 min followed by 40 cycles of denaturation at 95 °C for 15 s, annealing and extension at 60 °C for 1 min. Real time and endpoint fluorescence data was collected with an Eppendorf Mastercycler ep realplex 4 s (Eppendorf, Hamburg, Germany). Data was recorded as the mean C_t_ value normalized to the average of the housekeeping genes (ΔC_t_).

**Table 1:** List of mouse primer and probe sequences used for qRT-PCR

Gene Name	RefSeq No.	Exon Location	Primer	Probe
MEF2a	NM_001033713	5–6	5’-AAGTTCTGAGGTGGCAAGC-3’	5′−/56-FAM/TGCTGAATC/ZEN/ TGTCCTCCGAGAGTGG/3IABkFQ/− 3’
			5’-CTGATGCTGACGATTACTTTGAG-3’	
MEF2b	NM_001045484	2–3	5’-ATACTGGAAGAGGCGTTGC-3’	5′−/56-FAM/AATGTCGCA/ZEN/ GTCACAAAGCACGC/3IABkFQ/− 3’
			5’-CTAGACCAAAGGAACAGGCA-3’	
MEF2c	NM_025282	7–9	5’-GTTGCCGTATCCATTCCCT-3’	5′−/56-FAM/AGATCTGAC/ZEN/ ATCCGGTGCAGGC/3IABkFQ/− 3’
			5’-TGTAACACATAGACCTCCAAGTG-3’	
MEF2d	NM_133665	5–7	5’-TGACATAGCCATTCCCAACG-3’	5′−/56-FAM/CAGGCTCCA/ZEN/ TTAGCACTGTTGAGGT/3IABkFQ/− 3’
			5’-GCCAGCACTACAGAGAAACAG-3’	
Gapdh	NM_008084	2–3	5’-GTGGAGTCATACTGGAACATGTAG-3’	5′−/56-FAM/TGCAAATGG/ZEN/ CAGCCCTGGTG/3IABkFQ/− 3’
			5’-AATGGTGAAGGTCGGTGTG-3’	
Tbp	NM_013684	4–5	5’-CCAGAACTGAAAATCAACGCAG-3’	5′−/56-FAM/ACTTGACCT/ZEN/ AAAGACCATTGCACTTCGT/3IABkFQ/− 3’
			5’-TGTATCTACCGTGAATCTTGGC-3’

**Table 2:** List of human primer and probe sequences used for qRT-PCR

Gene Name	RefSeq No.	Exon Location	Primer	Probe
MEF2A	NM_005587	13–14	5’-GGTTCGGACTTGATGCTGAT-3’	5′−/56-FAM/AACCCTGAG/ZEN/ ATAACTGCCCTCCAG/3IABkFQ/− 3’
			5’-CACCACCTAGGACAAGCAG-3’	
MEF2B	NM_001145785	1–2	5’-GATGCGGGAGATCTGGATT-3’	5′−/56-FAM/CATCGTCCC/ZEN/ AGGCTGAGTGGAAT/3IABkFQ/−3’
			5’-CCCCTGATCTTCGTGCAG-3’	
MEF2C	NM_002397	3–4	5’-TGTTGGTGCTGTTGAAGATGA-3’	5′−/56-FAM/TGCTGTGTG/ZEN/ ACTGTGAGATTGCGC/3IABkFQ/−3’
			5’-AGATTACGAGGATTATGGATGAACG-3’	
MEF2D	NM_001271629	11–12	5’-CTGCACTGGTCAACTGGTAA-3’	5′−/56-FAM/CTCCCCTTC/ZEN/ TCTTCCATGCCCAC/3IABkFQ/−3’
			5’-CAGTCTACTCATTCGCTCACC-3’	
GAPDH	NM_002046	2–3	5’-TGTAGTTGAGGTCAATGAAGGG-3’	5′−/56-FAM/AAGGTCGGA/ZEN/ GTCAACGGATTTGGTC/3IABkFQ/−3’
			5’-ACATCGCTCAGACACCATG-3’	
TBP	NM_003194	5–6	5’-CAAGAACTTAGCTGGAAAACCC-3’	5′−/56-FAM/CACAGGAGC/ZEN/ CAAGAGTGAAGAACAGT/3IABkFQ/−3’
			5’-GATAAGAGCCACGAACCAC-3’	

### MEF2 isoform knockdown using siRNA

Accell SMARTpool™ siRNA constructs for knockdown of *MEF2a*, *MEF2b*, or *MEF2d* and Accell non-targeting control siRNA (siControl) were purchased from Dharmacon (Lafayette, CO). RENCA (7000/well) cells were seeded in 12-well plates (BD Biosciences, Franklin Lakes, NJ), allowed to attach overnight, and incubated with 1 μM siRNA in Accell delivery media for 72 H. Media and siRNA were replenished for an additional 72-h period to maximize gene knockdown. Following siRNA incubation, RENCA cells were evaluated for *MEF2a*, *MEF2b*, and *MEF2d* expression by qRT-PCR. Isoform expression was normalized to housekeeping genes and compared to untreated or siControl transfected cells to confirm knockdown.

### Tumor inhibitory capacity

The inhibitory capacity of RENCA macrobeads against freely growing RENCA, DU145 or DU145/GR cells was measured essentially as previously described [8, 13]. Briefly, RENCA (15,000/well), DU145 or DU145/GR (30,000/well) cells were seeded in 6-well plates (BD Biosciences), allowed to attach overnight, and cultured with or without RENCA macrobeads suspended in cell culture inserts (BD Biosciences). Following a 5-day incubation period, cells were methanol-fixed and stained with 0.33% (w/v) neutral red (Sigma-Aldrich, St. Louis, MO). The stain was extracted in 1 mL/well 1.25% (w/v) sodium dodecyl sulfate (Life Technologies) and absorbance read at 540 nm with 630 nm as the reference wavelength. Tumor inhibitory capacity (reported as percent inhibition) was defined as the percent difference in absorbance (540–630 nm) between treated and untreated media.

### In-cell western analysis

RENCA (90,000/cm^2^), DU145 or DU145/GR (150,000/cm^2^) cells were seeded in 96-well black, clear-bottom tissue culture-treated microplates (BD Biosciences), allowed to attach overnight, and synchronized using serum starvation. Cells were incubated with either naïve or 5-day conditioned media for 30 min, followed by pre-fixation in 2% paraformaldehyde (PFA; EMS, Hatfield, PA) for 10 min and fixation in 4% PFA for an additional 20 min at room temperature. The cells were permeabilized with 0.1% Triton X-100 in TBS (Sigma-Aldrich) followed by incubation in Odyssey Blocking Buffer™ (LI-COR Biosciences, Lincoln, NE) for 90 min at room temperature with gentle agitation. Cells were stained at 4 °C overnight with mouse monoclonal IgG antibody against EGFR (E-8) (SCBT, Dallas, TX) together with rabbit monoclonal IgG antibody against phosphorylated EGFR Tyr-1068 (EP774Y) (Millipore, St. Louis, MO) diluted in blocking buffer. In wells designated as no primary antibody controls, blocking buffer was added during the primary antibody incubation. Cells were washed with 0.1% Tween-20 in TBS (Sigma-Aldrich) and stained with donkey anti-mouse IgG IRDye™ 800CW and donkey anti-rabbit IgG IRDye™ 680RD (LI-COR Biosciences) for 1 h at room temperature protected from light. Images of target molecule fluorescence were obtained using the Odyssey Infrared Imaging System (LI-COR Biosciences). Integrated intensities of fluorescence in each well were quantified following subtraction of the average IR signal from wells designated as no primary antibody controls.

### Gefitinib treatment

Gefitinib (*N*-(3-chloro-4-fluoro-phenyl)-7-methoxy-6-(3-morpholin-4-ylpropoxy) quinazolin-4-amine) was purchased from Tocris (Minneapolis, MN). Adherent cells were pre-treated at the indicated concentrations of gefitinib or 0.01% DMSO for 2 h prior to application of either naïve or 5-day conditioned media containing the drug or vehicle control at the same concentration.

### Generation of drug resistant cell line

The gefitinib-resistant subline (DU145/GR) was established by culturing parental DU145 cells with incrementally increasing gefitinib concentrations from 1 μM to 3 μM over 6 months. DU145 cells were continuously maintained in gefitinib, with treatments beginning at the initial IC50 of the DU145 parental line [14]. Following recovery of doubling time compared to the parental DU145 cell line, the concentration of gefitinib was increased by 0.5 μM in DU145 culture media at each incremental step until gefitinib concentration was maximal at 3 μM. Assessment of resistance to gefitinib was performed every 4 passages for the first 12 passages and every passage thereafter. DU145/GR exhibited a 21-fold increase in resistance to the growth-inhibitory effect of gefitinib as determined by MTT assay, and the resistant phenotype had been stable for at least 6 passages under drug-free conditions prior to use in experiments. Parental DU145 cells that did not receive treatment were passaged alongside treated cells and were used at equivalent passage numbers.

### Statistical analysis

Statistical analysis was performed using Microsoft Excel software. Significant differences were analyzed using student’s *t* test and two-tailed distribution. Results were considered to be statistically significant if *p* < 0.05. Results were expressed as mean ± SD of at least triplicate experiments.

## Results

### Reporter arrays identify multiple pathways underlying RENCA macrobead function

**Fig. 1:**
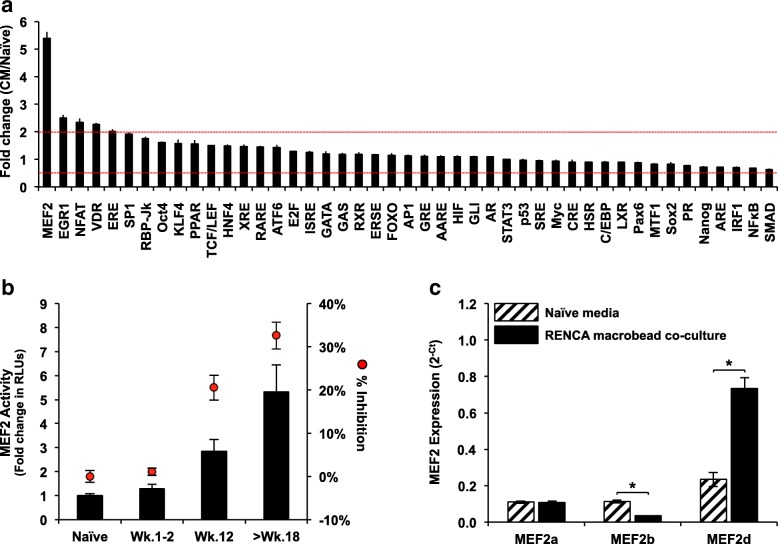
Factors secreted by RENCA macrobeads alter the transcription factor activity and expression of MEF2. (**a**) RENCA cells, transiently transfected with pathway-focused transcription factor-responsive luciferase reporter constructs were exposed to naïve media or 5-day conditioned media from > 18 wk. RENCA macrobeads. Fold-change, calculated based on normalized luciferase activity of the conditioned media (CM) response relative to the naïve media response, is graphed in descending order. Columns, mean (*n* = 3); bar, SD. Dotted lines at 2 and 0.5 on the y-axis indicate the threshold for two-fold up- and down-regulation respectively. (**b**) MEF2 reporter activity in RENCA cells exposed to 5-day RENCA macrobead-conditioned media. Reporter activity in response to naïve media was used as a control. Fold-change was calculated for each sample relative to the naïve media sample. Each column represents the mean (*n* = 6–8) ± SD (primary axis). Mean inhibitory response of RENCA macrobeads on freely growing RENCA cells; red circles (*n* = 3) ± SD (secondary axis). (**c**) RENCA cells were co-cultured with > 18 wk. RENCA macrobeads for 5 days. RENCA cells cultured in naïve media served as a control. Total RNA was isolated and subjected to real-time PCR analysis for the expression of *MEF2a*, *MEF2b*, *MEF2c* and *MEF2d*. *MEF2c* was not detected in RENCA cells. Each column represents the mean (n = 3) ± SD. *p < 0.001, compared with naïve media

To identify putative signaling pathways underlying the inhibitory response of RENCA macrobeads, the activity changes of 45 transcription factors associated with canonical signaling pathways were assessed in RENCA cells exposed to conditioned media from mature RENCA macrobeads. Based on pathway-specific transcription factor-responsive luciferase reporters, we identified 5 signaling pathways that were significantly activated in targeted tumor cells following conditioned media exposure (Fig. 1a, > 2 fold change, *p* < 0.05), with the highest transcription factor activity observed for MEF2.

Since the anti-proliferative effect of RENCA macrobeads increases over time following encapsulation, reaching a maximal inhibitory capacity at approximately 6 months [8], we sought to determine whether MEF2 activity in exposed tumor cells correlated with the age of the RENCA macrobeads. The MEF2 reporter construct was transiently transfected into RENCA cells and luciferase activity was measured following exposure to conditioned media from varied ages (1–2 week, 12 week and > 18-week) of RENCA macrobeads. In parallel with the increased inhibitory response of RENCA macrobeads, we observed an age-dependent increase in MEF2 activity as compared to naïve media (Fig. 1b).

### Exposure to RENCA macrobeads alters the expression of MEF2 isoforms

To identify specific MEF2 isoform(s) associated with RENCA macrobead-induced MEF2 activity, we assessed the expression of *MEF2a*, *b*, *c* and *d* in RENCA cells cultured in naïve media or together with RENCA macrobeads. RENCA cells expressed *MEF2a*, *MEF2b* and *MEF2d* isoforms in naïve media. Following exposure to RENCA macrobeads, *MEF2b* expression was reduced by 3.3-fold and *MEF2d* expression was increased 3.2-fold in RENCA cells, but *MEF2a* expression was unaffected (Fig. 1c).

### MEF2 expression is required for RENCA macrobead-induced inhibition

To determine whether the presence of MEF2 transcription factors in target cells contributed to the inhibitory effect of RENCA macrobeads, we used RNA interference to analyze the impact of individual isoforms of the MEF2 gene family. RENCA cells transfected with non-targeting control siRNA or siRNA directed against *MEF2a*, *MEF2b*, *MEF2d* or combined *MEF2a*, *MEF2b* and *MEF2d* (MEF2 pool) siRNAs were co-cultured with RENCA macrobeads using a cell culture insert system. Knockdown efficiency was confirmed to be greater than 80% by qRT-PCR and specific to the targeted isoform, at the beginning and at the end of the growth inhibition assay (day 0 and day 5, respectively; Additional file 1: Figure S1).

**Fig. 2:**
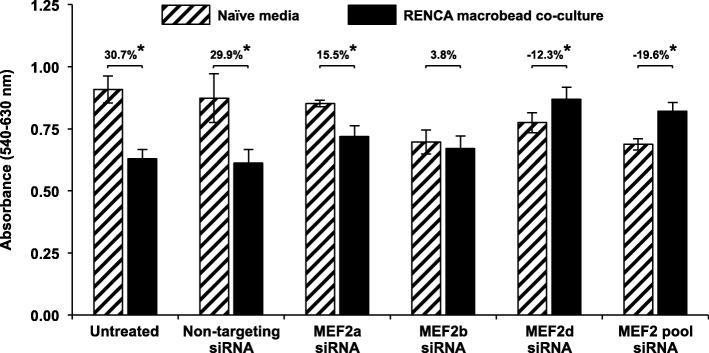
MEF2 is integral in mediating the anti-proliferative effect of RENCA macrobeads. RENCA cells were transiently transfected with two-rounds of 1 μM non-targeting siRNA, *MEF2a*, *MEF2b*, *MEF2d* or combined *MEF2a*, *b* and *d* siRNAs (MEF2 pool) for 72 h followed by culture in naïve media or together with > 18 wk. RENCA macrobeads in a cell culture insert system for 5 days. Each column represents the mean absorbance value (*n* = 3) ± SD following neutral red staining, with growth inhibition calculated as the percent difference in absorbance between the indicated conditions. *p < 0.005 compared with naïve media

Following co-culture with RENCA macrobeads, RENCA cells lacking siRNA treatment (untreated) exhibited 30.7% growth inhibition, similar to the growth reduction observed in RENCA cells transfected with non-targeting siRNA (29.9%). In cells lacking *MEF2a*, there was a significant decrease in inhibition as compared to cells treated with non-targeting control siRNA (15.5% vs. 29.9%) following exposure to RENCA macrobeads (Fig. 2). Silencing of *MEF2b* in combination with RENCA macrobead exposure did not reveal a substantial difference in the inhibitory response (3.8%). However, *MEF2d* knockdown promoted survival in response to factors secreted by RENCA macrobeads, increasing cell proliferation by 12.3% over baseline growth of cells exposed to naïve media, corresponding to a 42.2% (Non-targeting siRNA 29.9% + *MEF2d* siRNA 12.3%) growth increase over cells treated with non-targeting siRNA together with RENCA macrobeads. Combined siRNA-mediated knockdown of *MEF2a*, *MEF2b* and *MEF2d* in RENCA cells resulted in significantly reduced cell inhibition (49.5%; Non-targeting siRNA 29.9% + MEF2 pool 19.6%) following co-culture with RENCA macrobeads, suggesting a central role for MEF2 isoforms in coordinating RENCA macrobead-induced growth arrest of target RENCA cells (Fig. 2).

Independent of RENCA macrobeads, the knockdown of *MEF2a* and *MEF2d* did not influence cell growth in naïve media (2.4% and 10.2%, respectively) but gene silencing of *MEF2b* reduced proliferation by 20.3% in RENCA cells. Similarly, pooled *MEF2a*, *MEF2b* and *MEF2d* siRNAs suppressed growth of RENCA cells in naïve media by 21.3%, likely due to *MEF2b* knockdown (Fig. 2).

### RENCA macrobeads signal through the epidermal growth factor receptor (EGFR)

To understand how RENCA macrobead-secreted tumor-inhibitory proteins regulate MEF2 expression, we explored the activity of cell-surface receptors, specifically TGF-β/SMAD, BMP and EGFR, as they have previously been shown to converge on MEF2 transcriptional regulation. Evaluation of the phosphorylation status of the intracellular signaling components SMAD2 and SMAD1/5 by western blotting demonstrated minimal changes in target cell activity in response to RENCA macrobead exposure as compared to naïve media (data not shown), suggesting that signaling through TGF-β and BMP may not directly contribute to the inhibitory effect of RENCA macrobeads.

**Fig. 3:**
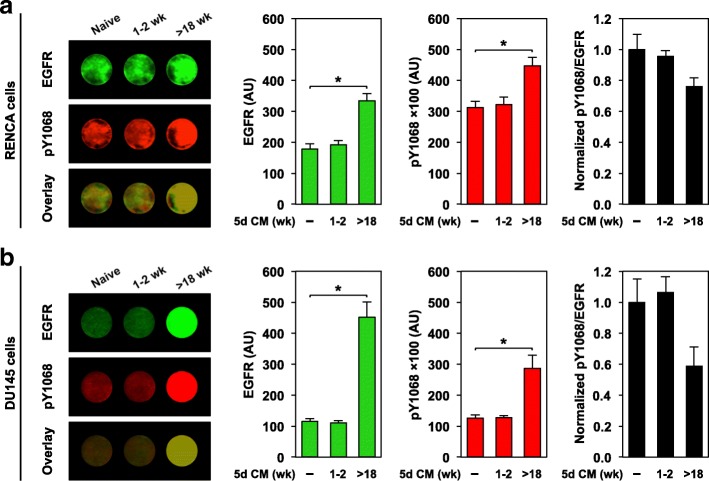
RENCA macrobeads regulate the epidermal growth factor receptor in both murine and human cell lines. In-cell Western analysis of total EGFR, phosphorylated EGFR (pY1068) and overlay (normalized pY1068/EGFR) in (**a**) RENCA cells, and (**b**) DU145 cells following exposure to 5-day conditioned media (CM) from RENCA macrobeads at 1–2 weeks or > 18 weeks of age as indicated. Cells cultured in naïve media were used as a control. Each column represents the mean normalized intensity in arbitrary units (AU) (*n* = 6–8) ± SD. *p < 0.001, compared with naïve media

In contrast, RENCA cells labeled with a monoclonal antibody that binds the cytoplasmic EGFR domain demonstrated a significant increase in EGFR abundance following exposure to conditioned media from mature (> 18 wk) RENCA macrobeads. In addition, phosphorylation of EGFR Tyr-1068 (pY1068), an indicator of EGFR activation, was increased in RENCA cells exposed to mature RENCA macrobead conditioned media. However, RENCA cells exposed to young macrobead (1–2 wk) conditioned media exhibited minimal changes in both EGFR and pY1068 levels (Fig. 3a).

To evaluate the relevance of signaling through EGFR in RENCA macrobead-mediated function, we first used an anti-EGFR monoclonal antibody (mAb528) that occludes the ligand-binding region of the mature EGFR ectodomain. However, phosphorylation of EGFR was unaffected by increasing concentrations of neutralizing antibody in RENCA cells (data not shown). Given the complexity of proteins secreted by mature RENCA macrobeads, mAb528 may not be present at a sufficient molar excess to effectively compete with potential EGFR ligands for access to the EGF receptor.

We next used the tyrosine-kinase inhibitor gefitinib to limit the activity of EGFR. Because the murine RENCA cells showed a limited response to gefitinib treatment, the human prostate cancer cell line DU145, previously shown to have a robust response to RENCA macrobead treatment [8] as well as gefitinib [14, 15], was used. MEF2 activity was confirmed in DU145 cells exposed to conditioned media from varied ages of RENCA macrobeads following transient transfection with the MEF2 reporter construct. Similar to the response observed in RENCA cells, a macrobead age-dependent increase in MEF2 activity was observed in DU145 cells in parallel with the increased inhibitory response of RENCA macrobeads (Additional file 2: Figure S2). A significant increase in EGFR abundance and elevated pY1068 EGFR was observed in DU145 cells following exposure to conditioned media from mature but not young RENCA macrobeads as compared to naïve media (Fig. 3b).

**Fig. 4:**
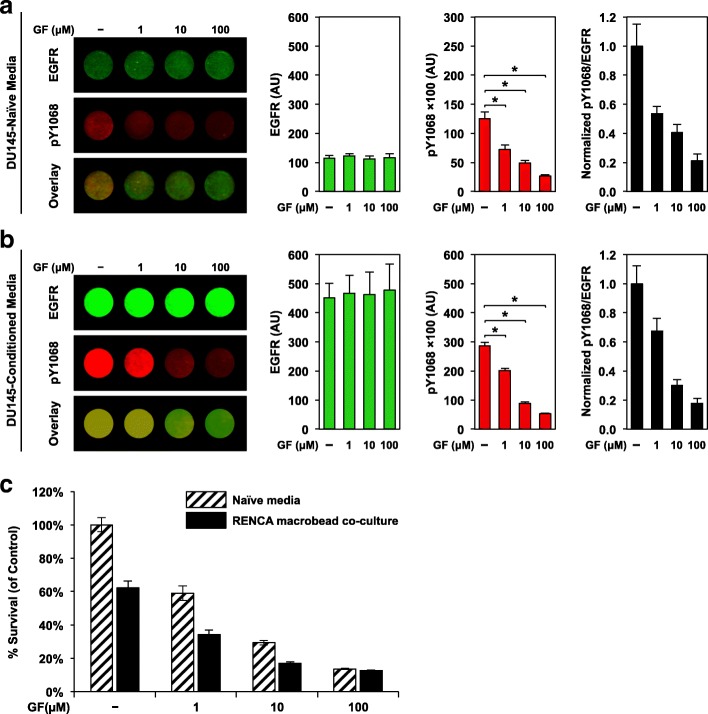
Gefitinib limits basal and RENCA macrobead-mediated EGFR activity and contributes to an additive inhibitory effect in DU145 cells. In-cell Western analysis of total EGFR, phosphorylated EGFR (pY1068) and overlay (normalized pY1068/EGFR) in DU145 cells pre-treated with gefitinib (GF) or 0.01% DMSO for 2 h at the indicated concentrations followed by culture in (**a**) naïve media, or (**b**) 5-day conditioned media from > 18 wk. RENCA macrobeads. Each column represents the mean normalized intensity in arbitrary units (AU) (n = 6–8) ± SD. *p < 0.001, compared with vehicle control. (**c**) DU145 cells pre-treated with gefitinib or 0.01% DMSO were cultured with naïve media or together with > 18 wk. RENCA macrobeads in a cell culture insert system for 5 days. Cells cultured in naïve media were used as a control. Histogram represents percent survival calculated as the percent absorbance of co-cultured samples relative to naïve media samples. Each column represents the mean survival (*n* = 3) ± SD

The capacity of gefitinib to counteract both basal and ligand (macrobead factor)-induced phosphorylation of the EGF receptor was verified, and subsequently cell proliferation of gefitinib-treated DU145 cells cultured with naïve media or mature RENCA macrobeads was assessed. Following culture in naïve media, in-cell western analysis revealed that gefitinib treatment exhibited minimal impact on the levels of total EGFR but predictably decreased Y1068 phosphorylation in a dose-dependent manner to below basal levels (Fig. 4a). A steady decrease in the level of phosphorylation was also observed in DU145 cells cultured with RENCA macrobead conditioned media (Fig. 4b); however, we observed higher baseline EGFR phosphorylation and EGFR expression, suggesting that RENCA macrobead mediated EGFR overexpression promotes autophosphorylation at basal levels. Gefitinib treatment exhibited an anti-proliferative effect similar to the growth inhibition observed following culture with RENCA macrobeads (Fig. 4c). Combination treatment with RENCA macrobeads and gefitinib inhibited tumor growth more efficiently than either treatment alone.

**Fig. 5:**
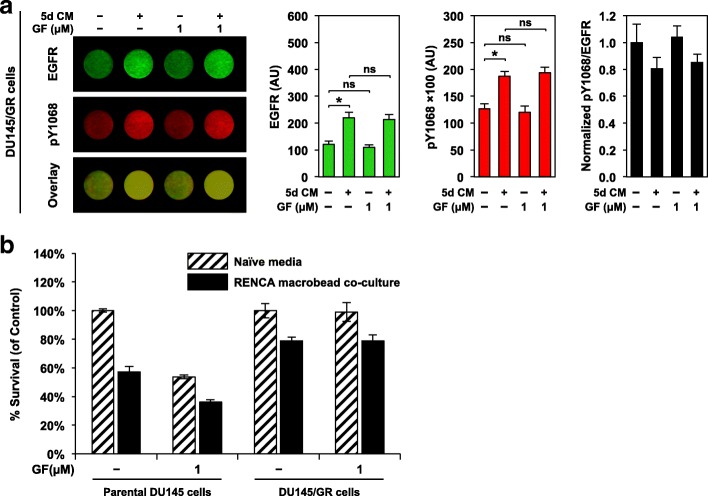
EGFR blockade results in partial attenuation of RENCA macrobead-mediated growth inhibition. (**a**) In-cell Western analysis of total EGFR, phosphorylated EGFR (pY1068) and overlay (normalized pY1068/EGFR) in DU145/GR cells pre-treated with 1 μM gefitinib (GF) or 0.01% DMSO followed by culture in naïve or 5-day conditioned media from > 18 wk. RENCA macrobeads. Each column represents the mean normalized intensity in arbitrary units (AU) (n = 6–8) ± SD. *p < 0.001, compared with vehicle control or conditioned media. (**b**) DU145/GR cells pre-treated with 1 μM gefitinib or 0.01% DMSO were cultured with naïve media or together with > 18 wk. RENCA macrobeads in a cell culture insert system for 5 days. Histogram represents percent survival calculated as the percent absorbance of co-cultured samples relative to naïve media samples. Each column represents the mean survival (*n* = 3) ± SD

To isolate the contribution of RENCA macrobeads to the enhanced inhibitory effect, we generated a gefitinib-resistant DU145 cell line. DU145/GR cells exhibited similar baseline EGFR expression and pY1068 EGFR levels as the parental DU145 cell line (EGFR: 121.03 ± 12.42 vs. 114.86 ± 9.51; pY1068: 12712.64 ± 908.77 vs. 12513.03 ± 1150.29 for DU145/GR and parental DU145 cells respectively) (Figs. 4a and 5a). Gefitinib treatment did not alter total or pY1068 EGFR levels (Fig. 5a) and had a nominal effect on the growth of DU145/GR cells (Fig. 5b). In response to conditioned media from mature RENCA macrobeads, we observed a moderate increase in EGFR abundance accompanied by a corresponding increase in EGFR phosphorylation (Fig. 5a). However, this response was significantly lower than the induction observed in the parental DU145 cell line by 2.04-fold and 1.53-fold respectively. Combined treatment with gefitinib and RENCA macrobead conditioned media exhibited an equivalent response of EGFR expression and phosphorylation as conditioned media alone. Furthermore, proliferation was reduced by 21.0% in response to conditioned media alone or in combination with gefitinib but was unaffected following treatment with gefitinib alone (Fig. 5b). This demonstrates that attenuation of the EGF receptor was associated with a 45.3% reduction in inhibition. Collectively, this suggests that RENCA macrobeads signal at least in part through the EGF receptor to modulate cell proliferation.

**Fig. 6:**
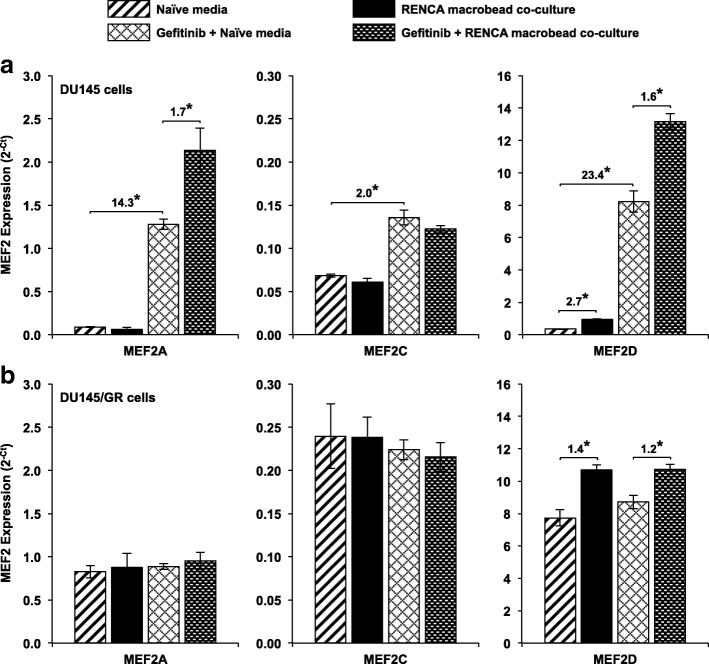
RENCA macrobeads modulate the expression of MEF2 isoforms through EGF receptor signaling. *MEF2A*, *MEF2B*, *MEF2C* and *MEF2D* expression was assessed by qRT-PCR on (**a**) DU145 or (**b**) DU145/GR cells cultured in naïve media or together with > 18 wk. RENCA macrobeads alone or pre-treated with 1 μM gefitinib followed by culture in media as described for 5 days. *MEF2B* expression was not detected in DU145 or DU145/GR cells. Each column represents the mean (*n* = 3) ± SD. *p < 0.005, compared with naïve media or gefitinib in naïve media

To further explore the molecular mechanism by which RENCA macrobead-mediated EGFR activation regulates cell proliferation, we examined MEF2 isoform expression in DU145 and DU145/GR cells exposed to RENCA macrobeads alone or in combination with gefitinib treatment. *MEF2A* and *MEF2C* expression in DU145 cells was not significantly altered when cultured together with RENCA macrobeads, whereas expression of *MEF2D* was increased 2.7-fold (Fig. 6a). *MEF2B* expression was not detected in DU145 or DU145/GR cells. Gefitinib treatment alone resulted in a significant increase in the expression of all expressed MEF2 isoforms (*MEF2A*: 14.3-fold; *MEF2C*: 2.0-fold; *MEF2D*: 23.4-fold) and combination gefitinib treatment with RENCA macrobead co-culture further elevated the expression of *MEF2A* (1.7-fold) and *MEF2D* (1.6-fold). Baseline expression of all MEF2 isoforms was significantly higher in DU145/GR cells as compared to the parental DU145 cell line (Fig. 6b). However, only *MEF2D* expression was significantly elevated following macrobead exposure alone (1.4-fold) or in combination with gefitinib treatment (1.2-fold).

## Discussion

In the current study, we provide evidence for a molecular mechanism linking the EGF receptor and MEF2 transcriptional regulation to the cell-growth-inhibitory response elicited by RENCA macrobeads. Taken together, our results support the idea that factors secreted by RENCA macrobeads activate the EGF receptor and modulate MEF2 expression to inhibit cellular proliferation. Attenuation of the EGF receptor resulted in approximately one-half of the cancer cell growth-inhibitory effect. These data suggest that the RENCA macrobeads exert their full growth inhibitory effects via more than the activation of a single receptor, at least with DU145 cells, and likely through more than one signaling pathway. These data also highlight the critical role of the MEF2 transcription factor in the inhibitory effect, especially the *MEF2D* and *MEF2B* isoforms, as knockdown of these isoforms resulted in a loss of growth inhibition with an increase in cell proliferation over control conditions.

MEF2s are pleiotropic transcription factors that contribute to oncogenic, as well as tumor suppressive, activities [16–18]. The finding that MEF2 transcription factors are integral to RENCA macrobead-induced growth inhibition in RENCA cells is consistent with the findings of other published studies. Knockdown of *MEF2b* in RENCA cells reduced cell proliferation, indicating that it may have oncogenic activity in this model system. A similar effect has been demonstrated in multiple diffuse large B-cell lymphoma cell lines, where knockdown of *MEF2B* led to down-regulation of BCL6 expression and repression of cell growth [19]. We have shown that RENCA macrobeads repress the endogenous expression of *MEF2b* in target RENCA cells.

The regulation of MEF2D is still controversial, with studies publishing both oncogenic and tumor suppressive capacities for this isoform [20, 21]. Exposure of target cells to RENCA macrobeads led to up-regulation of *MEF2D*. *MEF2d*-silencing using *MEF2d*-siRNA abrogated RENCA macrobead-mediated inhibition supports a tumor suppressive role in this model. In agreement with our observation, a recent study demonstrated that expression of exogenous MEF2D inhibited cell proliferation and anchorage-independent growth in rhabdomyosarcoma cell lines [22]. MEF2d has the capacity to regulate the transcription of G2/M transition-related genes. Upstream of the transcription start site, genes encoding GADD45 and CDKN1A/p21 contain putative MEF2 recognition elements [23]. MEF2D regulates the expression of these genes in a cell-context dependent manner [23, 24]. We have previously shown that *Gadd45* expression was elevated in RENCA monolayer cells treated with RENCA macrobeads [8]. Moreover, ectopically-expressed MEF2D can up-regulate p21, the cyclin-dependent kinase inhibitor that promotes cell-cycle arrest [22].

Although a direct effect on *MEF2a* expression was not observed in RENCA cells following culture with RENCA macrobeads, knockdown of *MEF2a* limited the ability of RENCA macrobeads to inhibit the growth of RENCA cells. Members of the MEF2 family bind as homo- and heterodimers to the appropriate DNA consensus sequence [25]. As a heterodimer, MEF2a preferentially interacts with MEF2d [26, 27]; as such, the loss of MEF2a could be at least partly responsible for the observed growth inhibition.

In addition, these studies support mechanisms of cancer growth regulation that extend beyond the prevalent notion of inhibiting oncogenic signals to achieve tumor inhibition. EGFR is popularly known for its pro-proliferation role as a growth factor receptor but this study illustrates that RENCA macrobeads effectively inhibit the growth of RENCA cells through an increase in EGFR-phosphorylation. Other studies have shown that both the levels of this receptor and the kinetic pattern of receptor engagement play important roles in the ultimate cell outcome. For example, She et al. have previously reported that the expression levels of EGFR and the duration of receptor activation are important for EGFR-mediated induction of apoptotic pathways [28]. In other studies, the intermittent activation of overexpressed EGFR inhibited cell death [29] while overexpression of EGFR accompanied by constitutive activation was associated with a pro-apoptotic effect [30]. As RENCA macrobeads continuously secrete factors, it is possible that sustained EGFR activation in both RENCA and DU145 cells contributes to receptor internalization and endosomal accumulation, leading to cellular apoptosis.

While these data demonstrate the critical role of the EGF receptor and MEF2 in the macrobead-induced growth inhibition, additional studies are required to investigate whether these molecules, with their distinct localizations and functions, are acting in concert with one another. Assessment of macrobead-induced regulation of MEF2 was attempted in HCT116 cells, a human colorectal cancer cell line, since RENCA macrobeads are currently being investigated in clinical trials as a treatment for metastatic colorectal cancer. Although *MEF2D* expression was increased more than 3.0-fold in HCT116 cells (Additional file 3: Figure S3) in response to co-culture with RENCA macrobeads, HCT116 cells exhibited low transfection efficiency with high cytotoxic effects. Furthermore, tyrosine kinase inhibitors (i.e. gefitinib) have been shown to be inactive in patients with colorectal cancer [31, 32], thus further investigations with this cell line were discontinued. Also, because blocking the EGF receptor in the murine RENCA cells proved difficult, as discussed in Methods above, only the human DU145 cell line was used to assess the effects of blocking this receptor. It remains possible that the EGF receptor does not play a role in the inhibition of RENCA cells, but this appears unlikely given the definitive phosphorylation of the receptor in the presence of the macrobeads. The role of EGF receptor and the MEF2 family of transcription factors in the RENCA macrobead-induced growth inhibitory effect could also apply to in situ tumors of varying origins and genetic profiles; we have observed tumor marker changes, decreases in 18F-fluorodeoxyglucose uptake by the tumors, and tumor necrosis during Phase I clinical trials using RENCA macrobeads as a treatment for numerous tumors, including prostate, colorectal, and hepatocellular carcinoma, among others [33].

An understanding of the mechanisms of action of the cancer growth inhibition produced by the RENCA macrobeads is critical to optimizing the therapeutic potential of this novel treatment. Phase 1 and 2 trials have recently been completed for treatment-resistant metastatic colorectal cancer [33–35]. Patients in these trials received RENCA macrobeads via outpatient laparoscopic implantation into the abdominal cavity. This methodology is thought to provide continuous release of macrobead-secreted peptides and proteins with tumor-inhibitory capacity [34, 35]. At least a 20% decrease in CEA and/or CA 19–9 in 75% of patients was observed with stable or decreased SUV in 35% of patients, thus this group of patients were classified as Responders. LDH levels remained stable and low in Responders (R) but increased steadily in Non-Responders (NR). Responders to RENCA macrobead implantation correlated with overall survival (OS): R mean OS = 10.76 mo.; NR mean OS = 4.9 mo.; *p* ≤ 0.0006. No serious adverse effects associated with the intraperitoneally implanted macrobeads were observed.

We have shown that the individual tumor colonies within the growth-restricted environment of the RENCA macrobeads regulate their own growth [36]. That is, the tumor colonies reach a maximal size and maintain this size through both cell death and growth processes. This remarkable display of tumor biology demonstrates that tumor growth can be controlled. Furthermore, the macrobeads’ ability to inhibit freely growing tumor cells external to the macrobead demonstrates that cancer cells can respond to regulatory signals provided by peptides, proteins and perhaps other modalities such as exosomes or various forms of RNA secreted from the macrobeads. It is our view that the RENCA macrobeads, as a complex biological system, act to provide a multifaceted systems restoration of normal function and interactions. As such, the macrobeads are not a precision-medicine approach to single targets. Rather, they can be considered as a biological- systems therapy that re-establishes more-normal regulation to a highly dysfunctional and dysregulated cancer system.

## Conclusions

Our studies reveal the involvement of a functional signaling pathway between RENCA macrobeads and EGFR/MEF2, where MEF2 plays an integral role in mediating anti-proliferative effects. These data support the hypothesis that the mechanism(s) of action of tumor growth inhibition induced by RENCA macrobead exposure operates through more than one cellular signaling pathway.

## Additional files

Additional file 1: **Figure S1.** MEF2 isoform expression in RENCA cells following transfection with MEF2 isoform-specific siRNA. Expression of *MEF2a*, *MEF2b* and *MEF2d* from RENCA cells transiently transfected with two-rounds of 1 μM non-targeting siRNA, *MEF2a*, *MEF2b*, *MEF2d* or combined *MEF2a*, *b* and *d* siRNAs (MEF2 pool) was assessed (a) at the beginning (day 0) of the growth inhibition assay and following culture for 5 days in (b) naïve media or (c) together with > 18 wk. RENCA macrobeads in a cell culture insert system. Untreated RENCA cells were included as a non-transfection control. Each column represents the mean (*n* = 3) ± SD. Relative expression is calculated as a ratio, with expression levels for the specified condition divided by the expression of the non-targeting siRNA for each isoform. (PDF 37 kb)

Additional file 2: **Figure S2.** Factors secreted by RENCA macrobeads alter the transcription factor activity of MEF2 in DU145 cells. MEF2 reporter activity in DU145 cells exposed to 5-day RENCA macrobead-conditioned media. Reporter activity in response to naïve media was used as a control. Fold-change was calculated for each sample relative to the naïve media sample. Each column represents the mean (*n* = 6–8) ± SD (primary axis). Mean inhibitory response of RENCA macrobeads on freely growing DU145 cells; red circles (n = 3) ± SD (secondary axis). (PDF 24 kb)

Additional file 3: **Figure S3.** RENCA macrobeads modulate the expression of *MEF2D* in HCT116 cells. *MEF2A*, *MEF2B*, *MEF2C* and *MEF2D* expression was assessed by qRT-PCR in HCT116 cells cultured in naïve media or together with > 18 wk. RENCA macrobeads for 5 days. *MEF2B* expression was not detected in HCT116 cells. Each column represents the mean (n = 3) ± SD. *p < 0.005, compared with naïve media. (PDF 23 kb)

## Abbreviations

DU145: Human prostate cancer cell line
DU145/GR: Gefitinib-resistant DU145 subline
EGFR: Epidermal growth factor receptor
FBS: Fetal bovine serum
FL: Firefly luciferase
GF: Gefitinib
MEF2 pool: Combined *MEF2a*, *MEF2b* and *MEF2d* siRNAs
MEF2: Myocyte-enhancer factor 2
NCS: Newborn calf serum
NR: Non-Responders
OS: Overall survival
PFA: Paraformaldehyde
pY1068: EGFR Tyr-1068
qRT-pCR: Quantitative real-time PCR
R: Responders
RENCA macrobeads: Agarose encapsulated murine renal adenocarcinoma cells
RIN: RNA Integrity Number
RL: Renilla luciferase
RLUs: Relative luciferase units
siControl: Accell non-targeting control siRNA
