# Supplementary figures and images for: MEF2 plays a significant role in the tumor inhibitory mechanism of encapsulated RENCA cells via EGF receptor signaling in target tumor cells
Source: BMC Cancer. 2018 Dec 4;18:1217. doi: 10.1186/s12885-018-5128-5 (PMC6280513; doi:10.1186/s12885-018-5128-5)

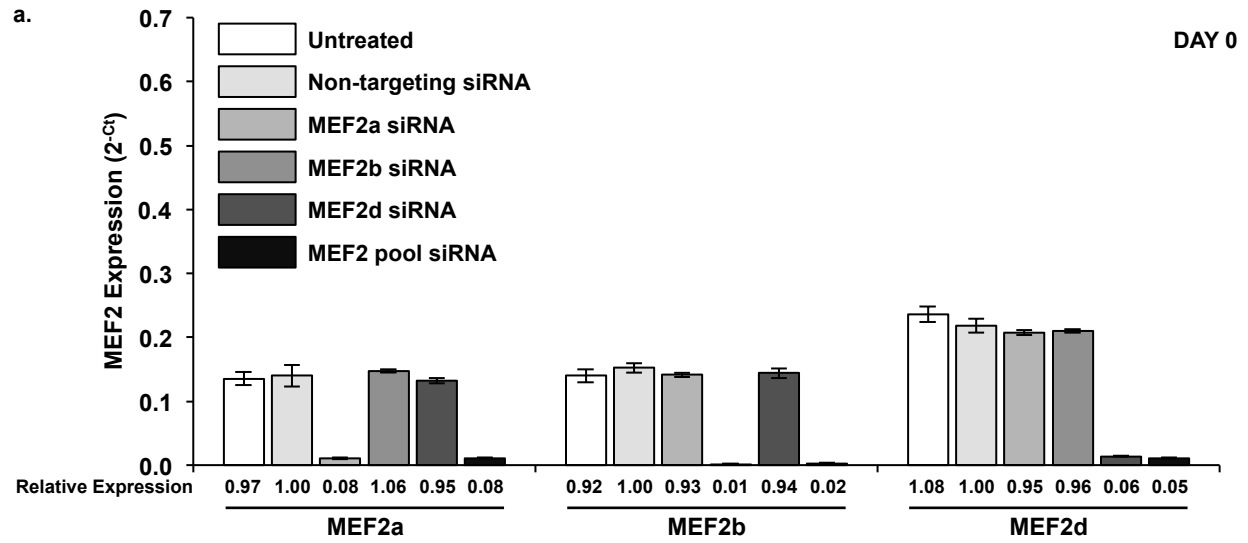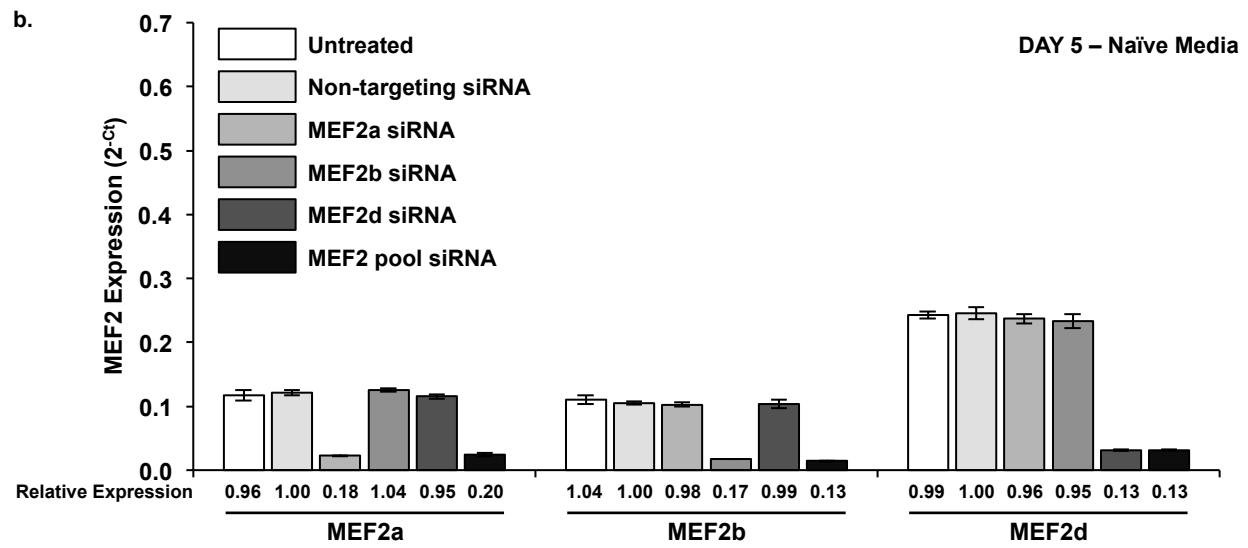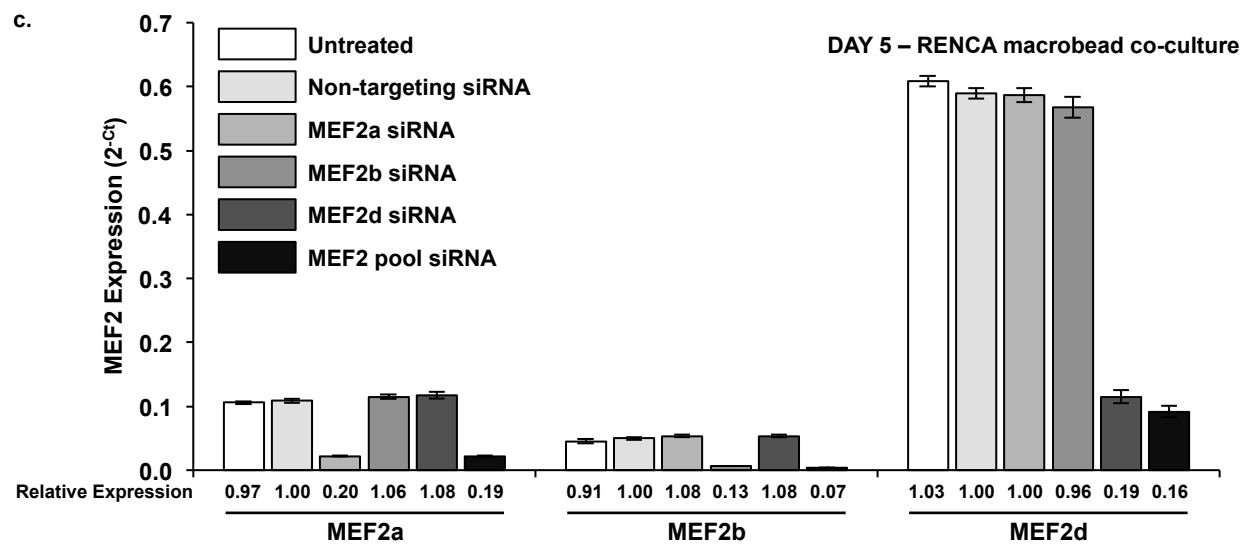

Supplement: Supplementary file 1 — Figure S1. MEF2 isoform expression in RENCA cells following transfection with MEF2 isoform-specific siRNA. Expression of MEF2a, MEF2b and MEF2d from RENCA cells transiently transfected with two-rounds of 1 μM non-targeting siRNA, MEF2a, MEF2b, MEF2d or combined MEF2a, b and d siRNAs (MEF2 pool) was assessed (a) at the beginning (day 0) of the growth inhibition assay and following culture for 5 days in (b) naïve media or (c) together with > 18 wk. RENCA macrobeads in a cell culture insert system. Untreated RENCA cells were included as a non-transfection control. Each column represents the mean (n = 3) ± SD. Relative expression is calculated as a ratio, with expression levels for the specified condition divided by the expression of the non-targeting siRNA for each isoform. (PDF 37 kb) [file 12885_2018_5128_MOESM1_ESM.pdf]

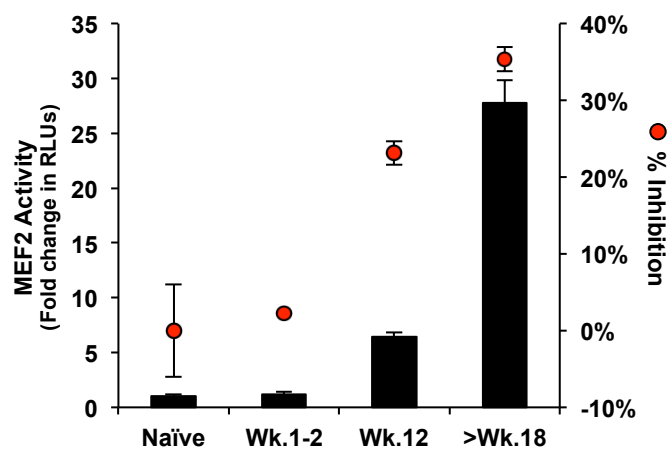

Supplement: Supplementary file 2 — Figure S2. Factors secreted by RENCA macrobeads alter the transcription factor activity of MEF2 in DU145 cells. MEF2 reporter activity in DU145 cells exposed to 5-day RENCA macrobead-conditioned media. Reporter activity in response to naïve media was used as a control. Fold-change was calculated for each sample relative to the naïve media sample. Each column represents the mean (n = 6–8) ± SD (primary axis). Mean inhibitory response of RENCA macrobeads on freely growing DU145 cells; red circles (n = 3) ± SD (secondary axis). (PDF 24 kb) [file 12885_2018_5128_MOESM2_ESM.pdf]

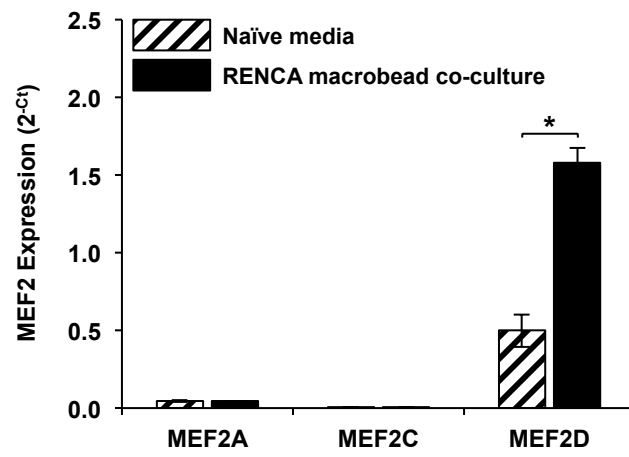

Supplement: Supplementary file 3 — Figure S3. RENCA macrobeads modulate the expression of MEF2D in HCT116 cells. MEF2A, MEF2B, MEF2C and MEF2D expression was assessed by qRT-PCR in HCT116 cells cultured in naïve media or together with > 18 wk. RENCA macrobeads for 5 days. MEF2B expression was not detected in HCT116 cells. Each column represents the mean (n = 3) ± SD. *p < 0.005, compared with naïve media. (PDF 23 kb) [file 12885_2018_5128_MOESM3_ESM.pdf]
